# Mitochondrial cellular organization and shape fluctuations are differentially modulated by cytoskeletal networks

**DOI:** 10.1038/s41598-023-31121-w

**Published:** 2023-03-11

**Authors:** Agustina Belén Fernández Casafuz, María Cecilia De Rossi, Luciana Bruno

**Affiliations:** 1 CONICET-Universidad de Buenos Aires, Facultad de Ciencias Exactas y Naturales, Instituto de Cálculo (IC), Buenos Aires, 1428 Argentina; 2 CONICET-Universidad de Buenos Aires, Facultad de Ciencias Exactas y Naturales, Departamento de Química Biológica, Instituto de Química Biológica (IQUIBICEN), Buenos Aires, 1428 Argentina; 3 Consejo Nacional de Investigaciones Científicas y Técnicas, Buenos Aires, Argentina

**Keywords:** Biophysics, Cell biology

## Abstract

The interactions between mitochondria and the cytoskeleton have been found to alter mitochondrial function; however, the mechanisms underlying this phenomenon are largely unknown. Here, we explored how the integrity of the cytoskeleton affects the cellular organization, morphology and mobility of mitochondria in *Xenopus laevis* melanocytes. Cells were imaged in control condition and after different treatments that selectively affect specific cytoskeletal networks (microtubules, F-actin and vimentin filaments). We observed that mitochondria cellular distribution and local orientation rely mostly on microtubules, positioning these filaments as the main scaffolding of mitochondrial organization. We also found that cytoskeletal networks mold mitochondria shapes in distinct ways: while microtubules favor more elongated organelles, vimentin and actin filaments increase mitochondrial bending, suggesting the presence of mechanical interactions between these filaments and mitochondria. Finally, we identified that microtubule and F-actin networks play opposite roles in mitochondria shape fluctuations and mobility, with microtubules transmitting their jittering to the organelles and F-actin restricting the organelles motion. All our results support that cytoskeleton filaments interact mechanically with mitochondria and transmit forces to these organelles molding their movements and shapes.

## Introduction

Mitochondria are dynamic organelles that experience size and morphological fluctuations due to fusion/fission events, transport, mechanical stresses, signaling cascades, among other processes that together affect their functionality^1,2^. Mitochondria interactions with other cytoplasmic components have been pointed out as factors that influence their dynamics^3–5^. Particularly, the cross-talk between these organelles and the cytoskeleton is of special interest because mitochondrial network structure, organization and function are affected by these interactions^6,7^.

The cytoskeleton is composed of microtubules, actin filaments (F-actin) and intermediate filaments (IFs) that form an interconnected network with characteristic biophysical properties and cell-type specific distribution^8,9^. This complex network is capable of exerting and transmitting mechanical forces to other cellular components like organelles, modifying their morphology and function. Generally, microtubules and actin filaments are mainly associated with transport phenomena mediated by molecular motors^10,11^, while intermediate filaments have support functions and resistance to mechanical stress^12–14^. Since mitochondria are associated with the three kinds of filaments in many eukaryotic cells, the cytoskeleton has emerged as a potential regulator of these organelles dynamics.

Microtubules are the main pathway through which mitochondria transport occurs. Dynein and kinesin motors anchor to mitochondria through adaptor proteins (e.g. Miro/Milton complex and KBP) and drive these organelles towards the microtubule’s minus and plus ends, respectively^15–18^. In addition, both microtubules and molecular motors can exert mechanical forces on these organelles too, promoting morphological and size variations. For instance, mitochondria have been shown to modify their size while being transported or can even be induced to deform by targeted recruitment of motors to their outer membrane^19–21^. Moreover, the role of microtubules and their associated proteins is also important in maintaining the balance between fission and fusion as well as mediating mitochondria tethering^22^.

On the other hand, actin filaments play a key role in mitochondria fission^23,24^. This complex process involves the division of one mitochondrion into two smaller ones and requires the joint action of cytoskeleton elements (e.g. F-actin and septin), organelles (e.g. endoplasmic reticulum) and specific proteins (e.g. myosin, dynamin-related protein 1)^1,25–28^ In addition, F-actin also has important functions in other processes such as mitophagy and mitochondria morphogenesis and inheritance. For instance, in the presence of mitochondrial damage, a rapid polimerization of actin filaments around mitochondria occurs, preventing the mitochondrial shape changes that take place upon their depolarization^29^. Besides, it has been proposed that Myosin 19—a motor associated to F-actin—would sculpt mitochondria cristae architecture acting as a mechanical tether that stimulates mitochondria morphogenesis^30^. Furthermore, different actin assemblies have been found to have complementary roles in the organization and partitioning of mitochondria in cell division; while a network of actin cables organizes mitochondrial positioning throughout the mitotic cytoplasm, a dynamic wave of actin filaments polimerize around mitochondria and form elongated comet tails that push the organelles randomly within the mother cell mixing healthy and damaged mitochondria between the daughter cells equitably^31^.

Finally, IFs influence mitochondrial function, subcellular localization and shape (see^32^ and references therein). Cytoskeletal IFs proteins vimentin and desmin have been reported to regulate mitochondrial bioenergetics in cardiac and skeletal muscle cells^33^. In particular, vimentin IFs have been shown to bind directly or indirectly to mitochondria through plectin, a cytolinker protein^34^, and to affect their mobility and anchoring within the cytoplasm^35,36^. Depletion of vimentin IFs resulted in the fragmentation of mitochondria in COS7 cells and it has been suggested that these filaments could participate in the mitochondrial association with microtubules^37^.

All this evidence points to a complex interaction of mitochondria and the cystoskeleton which is not completely understood. As previously mentioned, the cytoskeleton is the cellular mechanical element par excellence and is capable of propagating mechanical forces throughout the cell^38^. Particularly, the role that mechanical forces play in mitochondrial fission^39,40^, shape and organization^41^ has recently been highlighted.

In most cellular types mitochondria form an interconnected network exhibiting varied morphologies ranging from single vesicles to branching tubular structures in continuous remodelling. *Xenopus laevis* melanocytes are big ($\sim$ 50 $\upmu$m) and thin ($\sim$ 3$\upmu$m^42^) adherent cells that constitute an ideal system to study organelles organization, morphology and motion in a quasi 2D geometry^43,44^. Most of mitochondria within melanocytes display elongated shapes with widths ($\lesssim$ 0.35 $\upmu$m) that are typically smaller than their lengths in this cell line^19,45^, allowing the study of mitochondrial organization and dynamics from the single organelle to the cellular level.

In this paper, we explored the spatial and temporal interactions between fluorescently labeled mitochondria and cytoskeleton filaments in a *X. laevis* melanophores cell line stably expressing fluorescent microtubules. We selectively affected the different cytoskeletal networks (i.e. microtubules, F-actin and vimentin IFs) and studied their effects on mitochondrial organization, morphology and shape fluctuations. Cells were imaged using Airyscan super-resolution (SR) microscopy to characterize the chondriome, while confocal microscopy was used to study the shape temporal fluctuations and mobility of these organelles. Our results showed that the different cystoskeletal filaments have complementary roles in shaping mitochondria behavior. While microtubules play a prominent role in the organization and mobility of mitochondria, F-actin and vimentin IFs contribute to organelle morphology, underscoring the role of mechanical contacts in the shaping of these organelles. Our study reveals distinct properties of mitochondria-cytoskeleton interactions that could be relevant to understanding the relationship between mechanical signal transmission and mitochondrial function.

### Mitochondria distribution and local orientation rely on microtubules and the integrity of the cytoskeleton

**Figure 1:**
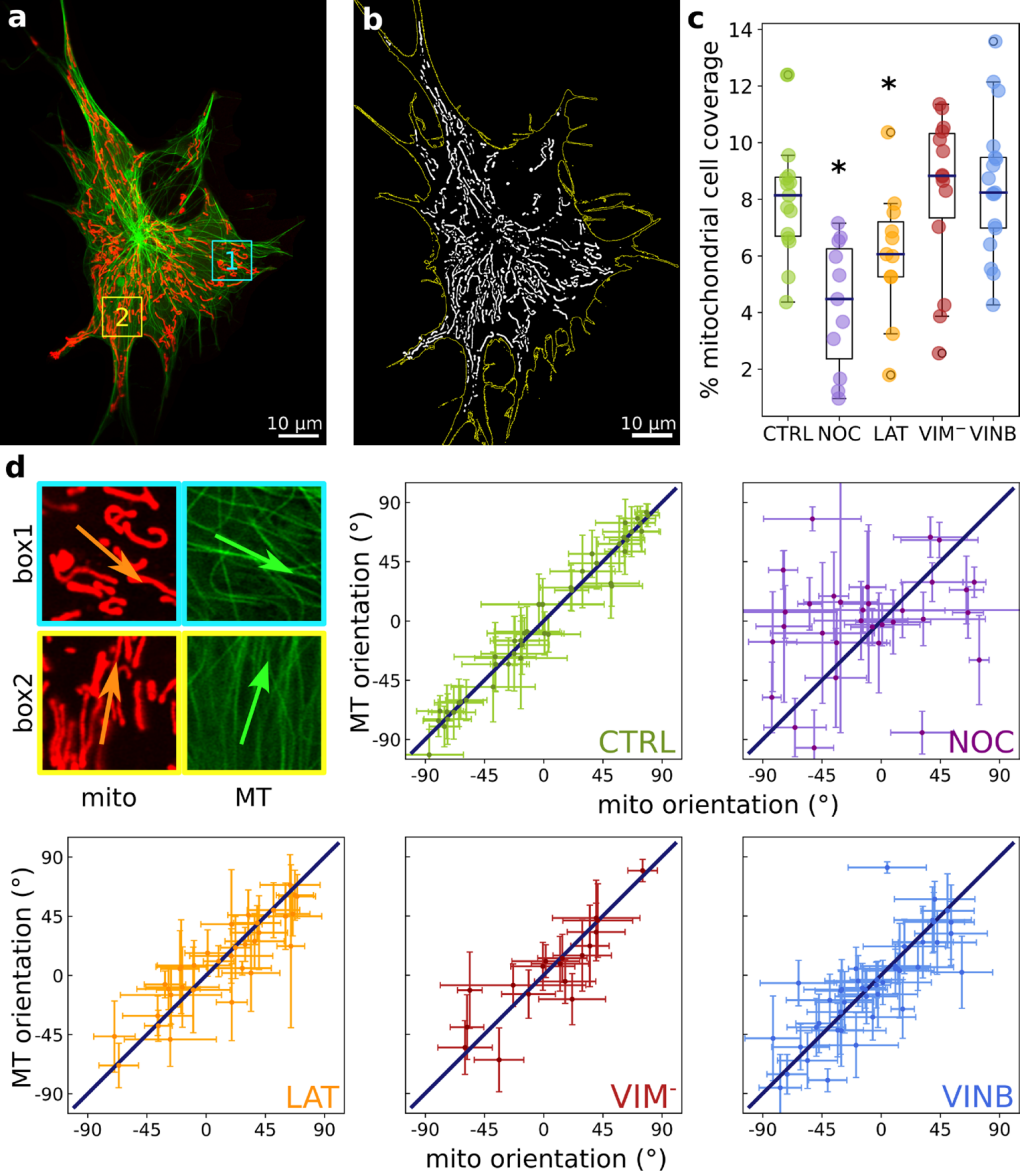
Dependence of mitochondrial network on the cytoskeleton integrity. (**a**) Representative Airyscan-SR image of *X. laevis* melanocyte expressing EGFP-XTP (green: microtubules) and incubated with MitoTracker Deep Red FM (red: mitochondria). (**b**) Binary image of the mitochondria network obtained for the cell shown in (**a**). The yellow line indicates the cell contour estimated as described in Supplementary Section 4. (**c**) Quantification of the mitochondrial cell coverage. Measurements were performed in control cells (CTRL), cells incubated with nocodazole (NOC), latrunculin-B (LAT) or vinblastine (VINB) and cells expressing the dominant negative vimentin mutant mCherry-(vim(1-138)) (VIM$^-$). Each circle represents a single cell. Asterisks denote significant differences (p-value < 0.05) with respect to the control condition. The values obtained are specified in Supplementary Table S2. (**d**) Mitochondria and microtubule orientation analysis. Zoom-in images of the regions included in the squares (box 1–2) delimited in the cell shown in (**a**). Mitochondria (mito) and microtubules (MT) images were analyzed independently to compute the local orientation angle (arrows) within the same subcellular region. Both magnitudes were plotted and compared through a linear regression routine as described in “Methods”.

We performed live-cell Airyscan-SR imaging of immortalized *X. laevis* melanocytes stably expressing the *Xenopus* homolog of $\tau$ protein XTP fused to EGFP^46^ and mitochondria labelled with MitoTracker Deep Red FM (Fig. 1a). To explore the roles that cytoskeletal networks play in the cellular organization of mitochondria, we selectively affected the different filaments that compose it. We used nocodazole to partially depolymerize microtubules, as assessed by preliminary calibration experiments (Supplementary Section 1 and Supplementary Fig. S1). We wanted to preserve part of the microtubule network because we aimed to explore more subtle aspects of mitochondrial dynamics and mobility that depend on microtubules. In addition, we analyzed the effects of vinblastine, a drug that stabilizes microtubules dynamics^47^. The concentration of vinblastine used in the experiments has the effect of increasing the curvatures of the microtubules, suggesting an increase in this network tensions^48^. Depolymerization of F-actin was performed incubating the cells with latrunculin-B as previously described^49,50^. Finally, transient transfection of melanocytes with a dominant negative vimentin mutant (vim 1-138) fused to mCherry resulted in the depletion of vimentin IFs network, as also previously studied in this cell line^42^. In summary, these treatments uncouple the different cytoskeletal filaments and, in particular, reconfigure the overall organization and morphology of the microtubule network, as represented in Supplementary Fig. S2.

Our experimental setup allowed us to explore and compare the distribution of mitochondria in control and cytoskeleton impaired cells (Supplementary Fig. S3). We used the Fiji plug-in Mitochondria Analyzer^51^ to quantify architectural features of the overall mitochondrial network (Fig. 1b; see “Methods”). We obtained different parameters for this characterization: the number, area and junctions of mitochondria, as well as the mitochondrial percentage of cell coverage (Supplementary Figs. S4 and S5).

When we analyzed the mitochondria ensemble behaviour we found that both, the average number of mitochondria per cell—around 145—and the area per mitochondrion—around 0.68 $\upmu$m$^2$— were similar for the different conditions (Supplementary Table S2). However, we observed a slight reduction of the number and size of mitochondria in nocodazole treated cells. The treatment with vinblastine also resulted in smaller organelles.

To further explore the mitochondrial cellular content, we computed the mitochondrial cell coverage cell by cell. This magnitude was defined as the ratio between the area of all mitochondria within a cell and its whole area, as described in “Methods”. We found that both latrunculin-B and nocodazole treated cells showed a reduction in cell coverage compared to control (Fig. 1c) indicating that mitochondria population is altered when F-actin or microtubule networks are disrupted and highlighting the crucial role these two cytoskeletal filaments play in mitochondrial homeostasis.

Finally, we analyzed the junctions per mitochondria, which quantify the ramifications observed in these organelles and found that around only 10% of the mitochondria present bifurcations (Supplementary Table S2). This result, added to the fact that the average length (see below) is about 6–7 times as long as the average width, confirmed the observation that most of the mitochondria in *X. laevis* melanocytes are curvilinear. Interestingly, while the fraction of forked mitochondria was reduced in cells treated with nocodazole, vinblastine enhanced this fraction, underscoring that microtubules organization and mechanical tensions influences mitochondria shapes.

Furthermore, a closer look at Fig. 1a reveals that most of the mitochondria appear to be aligned with neighboring microtubules, suggesting that these cytoskeleton filaments may act as a template for mitochondrial organization. Hence, we computed the local orientations of mitochondria and microtubules in 10 $\times$ 10 $\upmu$m$^2$ patches on Airyscan-SR images of control cells, as described in “Methods”, and compared both magnitudes (Fig. 1d). We found a strong correlation between microtubules and mitochondria directionality with the data aligned along the identity line (RMSE: 8.95$^{\circ }$, Fig. 1d).

To rule out that other cell structures would be responsible for this co-alignment, we explored cell regions expressing both mitochondria and microtubules in nocodazole-treated cells and performed the co-orientation analysis (see “Methods” for details). Notably, the orientations correlation was lost (RMSE: 54.7$^{\circ }$, Fig. 1d), revealing that the microtubule network functions as a mitochondrial scaffolding in this cell line.

We next wondered if the other cytoskeletal networks play a role in the microtubules dependent mitochondrial organization. Thus, we analyzed the co-orientation of mitochondria and microtubules in latrunculin-B treated cells and in cells expressing mutant vimentin, finding that the co-orientation was weaker in those cells as indicated by the larger dispersion of the data around the identity (RMSE: 18.1$^{\circ }$ and 18.5$^{\circ }$, Fig. 1d).

Although our data suggest that the three cytoskeleton filaments are involved in the cellular organization of mitochondria, the results showed that microtubules have the largest effect on these organelles’ layout. We wondered if this task relies on microtubules stability, so we treated cells with vinblastine and repeated the analysis. The co-orientation of mitochondria and microtubules was also weaker in this condition (RMSE: 23.3$^{\circ }$, Fig. 1d), indicating that microtubules dynamics plays an important role in mitochondrial docking or that stress accumulated in these filaments affects the cross-linking between the two structures.

The results showed that although mitochondrial organization and local orientation are strongly correlated with microtubules, this co-alignment is partially impaired by cell treatments that depolymerize or disrupt other cytoskeletal networks, or affect microtubule dynamics, indicating that mitochondria positioning relies on the integrity of the cytoskeleton.

### Different cytoskeletal networks scaffold the shape and curvature of individual elongated mitochondria in distinct ways

As in many other cellular systems, mitochondria in *X. laevis* melanocytes display different shapes: rounded, elongated (simple or forked) and ringed. From all these, simple elongated mitochondria represent the most common organelles in this cell line, as assessed in the previous section, and thus can be approximated as curvilinear filaments.

Previous studies showed that the analysis of the curvatures of cytoskeletal filaments provides information on the mechanical properties of these filaments in cells and their mechanical crosstalk with other components^48,52–54^. The same was shown for endoplasmic reticulum tubules^55^. Based on these results, we propose that the shapes of mitochondria could provide clues on the mechanical properties of these organelles and their interaction with the cytoskeletal filaments.

**Figure 2:**
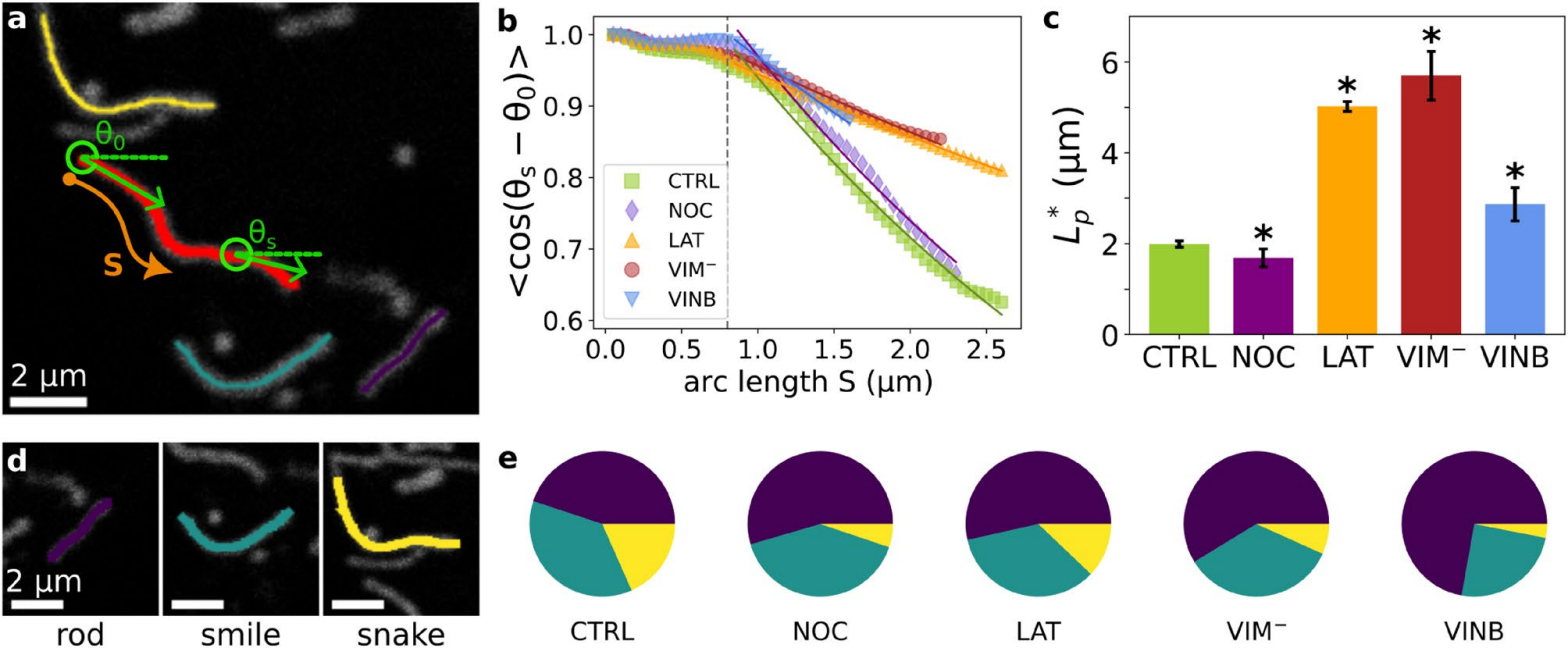
Characterization of mitochondria apparent stiffness and shape distribution. (**a**) Representative confocal image of mitochondria within melanocytes. Elongated mitochondria showing characteristic shapes (shown in colours) were analyzed to compute the tangent angle ($\theta$, green) at a distance *s* (orange arrow) from their edge. (**b**, **c**) Determination of mitochondria apparent persistence length ($L_p^*$). The ensemble average of the correlation of the tangent angle obtained for each experimental condition was fitted with Eq. (5) to determine the $L_p^*$ (**b**). $L_p^*$ values are shown in (**c**) and Supplementary Table S5. The values were considered significantly different with respect to the control condition (indicated by asterisks) if the error intervals did not overlap, as described in “Methods”. (**d**,**e**) Distribution of mitochondria shapes. Mitochondria shapes were classified as rod- (violet), smile- (light blue) and snake-like (yellow) according to their curvature (**d**). The proportion of these mitochondrial populations obtained for each experimental condition are illustrated in (**e**) and displayed in Supplementary Table S5.

Following this idea, we used Fiji plug-in JFilament^56^ to recover the spatial coordinates $\{x,y\}$ of individual elongated mitochondria from confocal microscopy images as described in “Methods”. These coordinates were then transformed to $\theta (s)$ representing the tangent angle of the curvilinear shape at a distance *s* from the edge (Fig. 2a).

First, we computed the length of the organelles (*L*) integrating the curvilinear coordinate *s* along the recovered shapes, as explained in “Methods”. We compared the distributions of lengths obtained for a large number of mitochondria (Supplementary Tables S3–S4 and Supplementary Fig. S6) and found that mitochondria were longer in latrunculin-B treated cells compared to control, as previously reported^24^. It has been shown that the depolimerization of F-actin results in a decrease of mitochondrial fission rate resulting in an increase in their length^57^, in agreement with our results. On the other hand, the treatment with vinblastine produced shorter mitochondria. We speculate that since vinblastine has been reported to increase microtubules mechanical stress^47,48^, this tension may be transmitted to mitochondria and would increase the fission rate^40^ resulting in shorter organelles. Finally, no differences were found for mitochondria lengths between control and nocodazole treated cells or cells expressing the vimentin mutant.

Considering the subset of long mitochondria with lengths larger than the population median, we quantified the apparent persistence length $$L_p^*$$^58^ in order to estimate the bending elasticity of these organelles in living cells as described in “Methods” and in a previous work^45^. Figure 2b shows the ensemble correlation of mitochondria curvature for the different treatments. As already pointed out in a previous work^45^, the finite width of mitochondria imposes a lower limit to the bending of mitochondria. Taking this into account, we were able to fit an exponential decay function for $$s>$$ 0.8 $$\upmu $$m obtaining the $$L_p^*$$ values from this fit.

The apparent persistence length of mitochondria in control cells was around 2 $\upmu$m (Fig. 2c and Supplementary Table S5), as also obtained in our previous work^45^. The partial depolymerization of microtubules with nocodazole did not affect significantly the $L_p^*$ value presumably indicating that long mitochondria were probably associated to the remaining polymerized microtubule network. On the other hand, the depolimerization of F-actin or the disruption of vimentin IFs significantly increased the apparent persistence length of mitochondria showing that that both cytoskeletal filaments mold this organelle shape probably adding mechanical contact points that deform the organelles resulting in more curved shapes. A similar result is obtained for cells treated with vinblastine: mitochondria showed an increment of their persistence length in comparison to control revealing that the stability of microtubules also contributes to the mitochondria shapes.

Next, we explored the ensemble distribution of mitochondria shapes. To this end, the shapes of mitochondria were classified as rod-, smile- and snake-like by means of an automatic classifier based on the Fourier decomposition of the curvature and previously developed by our group^45^ (Fig. 2d; see “Methods” for details). We found that all treatments enhanced the population of rod-like mitochondria in detriment of more curved ones (Fig. 2e and Supplementary Table S5), reinforcing a picture where the different filaments constitute a complex, dynamic and integrated network where mitochondria nestle.

All these results suggest that the different cytoskeletal networks finely tune the lengths and shapes of mitochondria in cells by providing mechanical stress and contact points to the organelles.

### Mitochondria shape fluctuations and mobility depend on microtubule and actin filament networks

In order to determine whether the cytoskeleton would also modulate differently the rapid fluctuations of mitochondria shapes, we tracked individual organelles from confocal image sequences and recovered their shapes and average positions. The temporal window used in these experiments (from seconds to minutes) and the explored spatial regions ($\sim$ 10 $\times$ 10 $\upmu$m$^2$) allowed us to capture fast mitochondria shape and movements variations.

**Figure 3:**
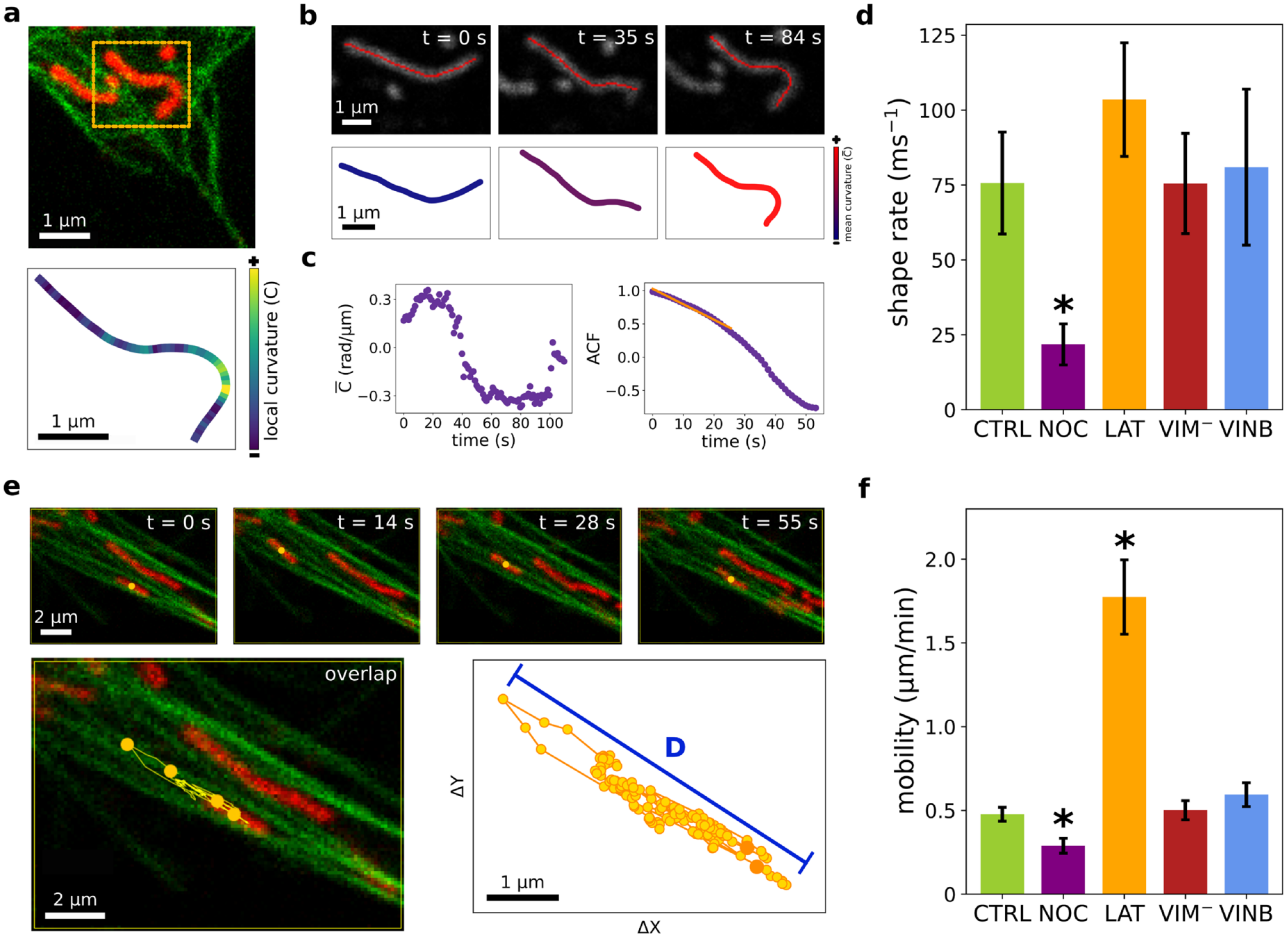
Mitochondria shape fluctuations and mobility. (**a**–**c**) Quantification of mitochondria curvature variation. Example of a mitochondrion shape and local curvature (*C*) along its length (a). The mean curvatures ($\overline{C}$) determined at each frame of the time-stacks (**b**) were used to calculate the ACF (**c**) as described in “Methods”. ACF data was fitted with an exponential decay function to obtain the characteristic time. (**d**) Mitochondria curvature fluctuation rate. The characteristic decaying time obtained from ACF analysis was used to compute the organelles shape fluctuation rate for each experimental condition. (**e**) Mitochondria mobility analysis. Representative time-lapse images of a moving mitochondrion (top panel). The spatial coordinates of the organelles (red) were recovered in each frame of the movies to obtain the trajectory of its center of mass (orange circles), (bottom panel). The start and end point of the trajectory are shown in dark orange. The maximum displacement of the organelle (D) is schematized as a blue segment. (**f**) Quantification of mitochondria mobility. Asterisks denote significant differences (p-value < 0.05) with respect to the control condition. Supplementary Table S6 displays the values shown in (**d**,**f**).

Temporal behavior of mitochondria morphological fluctuations was quantified by computing the mean curvature of each mitochondrion at frame *i* (Fig. 3a,b) as the average tangent angle derivative with respect to *s* (see “Methods” for details). Then, we calculated the auto-correlation function (ACF) over time of the mean curvature. Most of the data decayed exponentially; thus, a characteristic time was recovered from the exponential fit of the ACF, as illustrated in Fig. 3c. We determined the rate of the curvature fluctuations as the inverse of these characteristic times (Fig. 3d). Our results showed that when the microtubule network was affected by nocodazole, mitochondrial fluctuations slowed down, supporting that microtubules are primarily responsible for rapid changes in mitochondria shapes.

In this way, we hypothesize that microtubules would transmit their jittering to mitochondria by means of the close associations between these two cellular components. This hypothesis agrees well with previous data showing that the characteristic times of lateral movements of microtubules in this cell line are between 10 and 100 s^59^, which are similar to the temporal window considered here. To test this hypothesis, we simultaneously tracked the transversal motion of mitochondria and neighboring microtubules in control cells and found a strong correlation in their lateral movement (Supplementary Figs. S7 and S8 and Supplementary Videos S1–3) suggesting the presence of close mechanical contact between microtubules and mitochondria.

We next wondered whether the shape fluctuations of individual mitochondria were reflected in the net motion of these organelles. So as to further explore mitochondrial movements, we recovered the trajectories of the position of the center of mass of individual organelles along the movies and measured the maximum distance (D) obtained for this magnitude, as schematized in Fig. 3e (see details in “Methods”). Representative trajectories of mitochondria in the different conditions are displayed in Supplementary Fig. S9 and Supplementary Videos S4–8. Mitochondria mobility was obtained by dividing D by the duration of the trajectory, and the results are shown in Fig. 3f. We found that individual mitochondria in nocodazole-treated cells exhibited more confined trajectories than in control cells, while mitochondria mobility increased in cells where F-actin network has been disrupted, following the trend observed for shape fluctuations. No significant differences were observed for mitochondrial mobility between control and vimentin IFs disrupted cells or cells treated with vinblastine.

Our results reveal distinct roles played by the different cytoskeletal filaments in mitochondrial motion and docking. Microtubules are known to be fundamental tracks for mitochondrial transport^7^ and thus it is expected that active motion would be impaired with nocodazole treatment. Furthermore, it has been suggested that IFs play a role in bringing mitochondria closer to microtubules and serve as an anchorage site for mitochondria reducing mitochondrial transportation^35^; consequently, an increase of mitochondria mobility would be expected in vimentin-disrupted cells. However, we did not observed this effect in our experiments. Finally, it has been shown that F-actin wrapped mitochondria during actin cycles in metaphase HeLa cells^24^, probably restricting mitochondria motion. In addition, the disruption of F-actin by latrunculin-B has been reported to result in increased mitochondrial motility, while enhanced polymerization of actin blocked fast movements of mitochondria in CV-1 cells^60^. Based on this evidence, we speculate that F-actin would attenuate the mobility of mitochondria in control cells. Our hypothesis is that this attenuation can be explained by a greater spatial confinement produced by the F-actin network in control conditions, which is obviously reduced after depolymerization of these filaments by latrunculin-B.

Although most of the organelles move modestly in the observation times, some very processive trajectories of rod-like mitochondria were observed (Supplementary Videos S9–10), as described in previous works^20,45^. Since the frequency of these events is low in the temporal window analyzed here, we have not done a statistical analysis of their dependence with the different treatments, which is left for future work.

## Discussion

**Figure 4:**
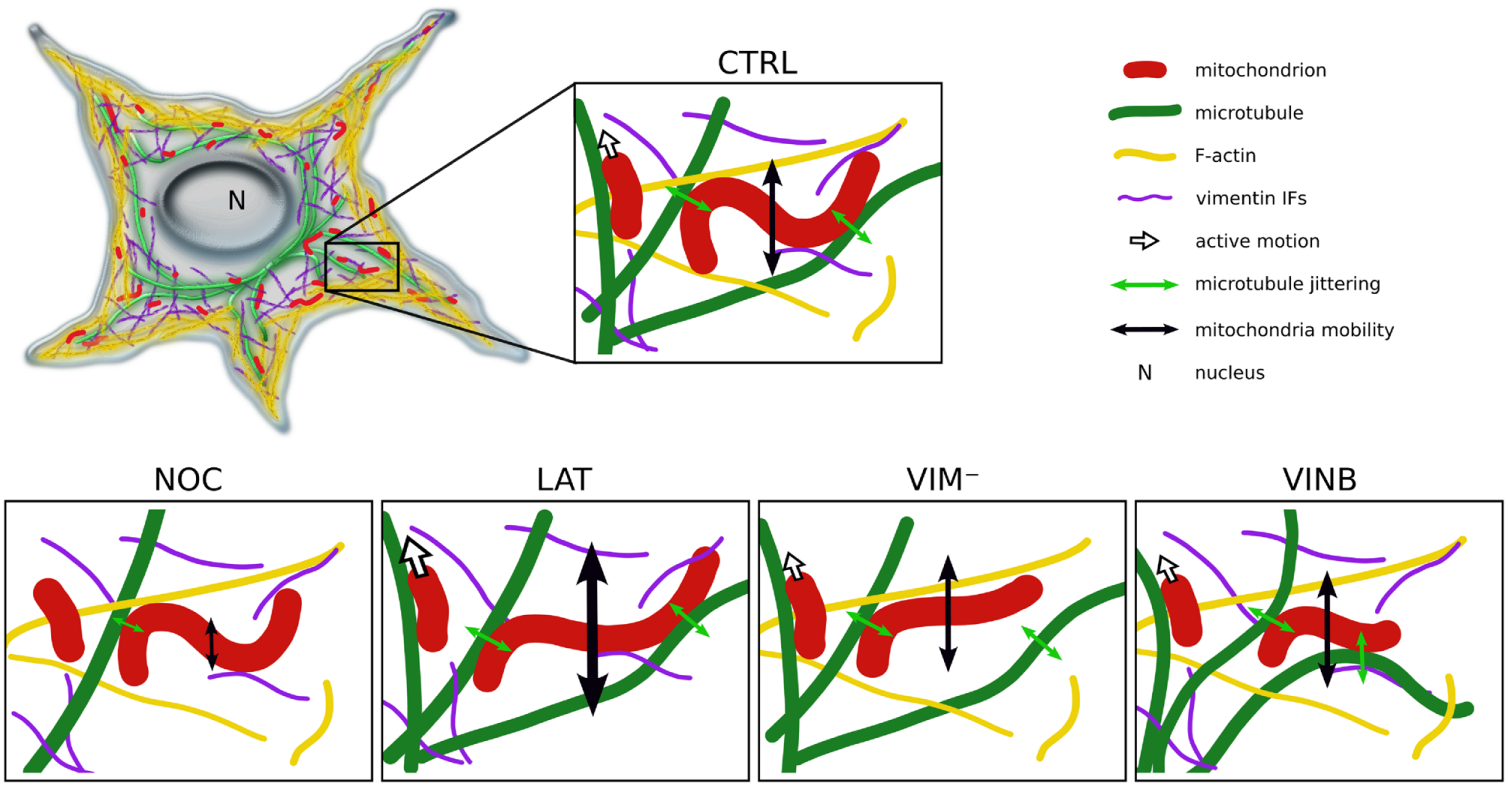
Summary of the modulation of mitochondrial shape fluctuations and mobility by the cytoskeleton. Mitochondria are in close association with microtubules, being transported through them and modifying their shape as a consequence of the jittering transmitted by these filaments (green double arrows) and the interactions with F-actin and vimentin IFs, both of which would contribute to maintain mitochondria confined to microtubule network. Upon partial depolymerization of microtubules (NOC), both the mobility of the organelles (schematized with the black double arrows) and the mechanical force imposed on them decrease. Given the disruption of F-actin (LAT) and vimentin IFs (VIM$^-$) networks, a predominance of elongated mitochondria is observed, suggesting that these filaments also modulate the organelles’ shape. F-actin depolymerization also results in increased mitochondrial mobility, suggesting that these filaments impose greater spatial confinement that restricts their motion. Perturbation of microtubule dynamics (VINB) decreases mitochondrial curvature and length compared to the control condition (All images created by A.B. Fernández Casafuz).

In this work, we explored the cross-talk between mitochondria and the cytoskeleton in living cells by selectively affecting the different cytoskeletal filaments (microtubules, F-actin and vimentin IFs). Our results showed that cytoskeleton perturbation has a differential impact on mitochondria length, apparent rigidity, mobility and cellular organization within *X. laevis* melanocytes. Our main results at the single-organelle level are summarized in the illustration shown in Fig. 4.

As previously reported in other cell lines^61^, the greatest effect was found by the partial depolymerization of microtubules with nocodazole, resulting in substantial changes in mitochondria cellular distribution and mobility. Microtubule network scaffolds mitochondrial organization in cells, as evidenced by the misalignment between these organelles and neighboring microtubules in nocodazole-treated cells. Moreover, mitochondria cellular distribution is reduced in this condition, probably due to impairment in intracellular transport caused by the disruption of the microtubule network. At the single-organelle level, mitochondria mobility and shape fluctuations are significantly reduced in this condition suggesting the presence of local transient associations between them and microtubules. These tethering events would transmit mechanical forces to the mitochondria, as shown by the correlation of the lateral movement of these organelles and microtubules in control cells.

On the other hand, F-actin and vimentin IFs would help keeping the mitochondria confined to the microtubule network, since their disruption results in a reduction of both structures co-orientation. At the same time, the increase in the apparent persistence length of mitochondria in F-actin and vimentin depleted cells also indicates that these two cytoskeleton filaments mechanically communicate with mitochondria, as reported^6,32^. These contact points would modulate mitochondria shapes resulting in more curved organelles.

Furthermore, not only the disruption of F-actin results in longer mitochondria, probably because of a decrease in their fission rate, as previously reported^24^, but also, their mobility is significantly enhanced, in agreement with previous studies^60^. For instance, in *Drosophila* neurons it has been shown that myosin V depletion produced an increase in mitochondrial length and a reduction of mitochondria motion^62^. Since myosin V tethers to actin filaments, the disruption of this network with latrunculin-B might have similar effects in the cell line studied here.

It is interesting to note that nocodazole and latrunculin-B treatments have opposite effects on mitochondria curvatures and mobility, highlighting the complementary roles these cytoskeletal networks play in these organelles spatio-temporal behavior. While microtubules constitute a dynamic mesh for the organization of mitochondria, transmitting mechanical impulses to them, F-actin and, to a lesser extent, vimentin filaments, restrict their movement and keep them in close association with the microtubule network.

Complementing these observations, we found shorter mitochondria in vinblastine-treated cells compared to control condition. In view of the reports of vinblastine enhancing microtubules curvatures^47,48^, we hypothesized that the extra mechanical loading, due to the accumulated tension in microtubules, would be transmitted to mitochondria. As a consequence, this mechanical stress would increase the mitochondria fission rate, as recently reported^40^.

Although we cannot rule out that changes in the organization of microtubules after the different treatments would contribute to some of the differences observed in mitochondrial organization and dynamics, our results reinforce the complementary roles of the cytoskeletal networks in shaping the cellular organization, morphology and mobility of these organelles. For instance, the fact that mitochondria mobility is enhanced after the disruption of the F-actin network (Fig. 3) is difficult to be explained only by differences in the global organization of microtubules. Moreover, the reduction in mitochondria-microtubules coalignment in F-actin and vimentin depleted cells (Fig. 1) is better understood considering that these filaments interact with mitochondria themselves, as previously reported^2,6,7,32^, and not only mediated by microtubules. Nevertheles, to achieve a better comprehension of how these mechanical cues impact on mitochondria function and dynamics more works are still required in this field of research.

## Methods

### Cell culture

Immortalized *Xenopus* *laevis* melanophores stably expressing the *Xenopus* tau-like protein XTP fused to EGFP^43,46^ were cultured as described in^63^. This cell line was kindly provided by Dr. Vladimir I Gelfand (Northwestern University, Chicago, IL). Cells were grown in 70% L-15 medium (Sigma-Aldrich) supplemented with bovine fetal serum. Phenylthiourea (PTU) was used to reduce the number of melanosomes^49^. For microscopy measurements, cells were grown for 2 days on sterilized 25 mm round coverslips placed into 35 mm dishes in 2.0 ml of complete medium.

### Plasmids and transfection

Transient transfection was carried out using Lipofectamine 2000 (Invitrogen) following the vendor instructions and observed 24 h after transfection. Cells grown on coverslips were transfected with the dominant-negative construct containing the head and alpha-helical domain 1A of vimentin fused to the red fluorescent protein [mCherry-vim(1-138)]. The plasmid was generated from the GFP-vim(1-138)^64^ that was kindly provided by Dr. Vladimir I Gelfand (Northwestern University, Chicago, IL).

### Sample preparation for imaging and conditions for drug treatment

For microscopy measurements, cells were plated onto 25 mm round coverslips as described above. Before observation, the coverslips were mounted in a custom-made chamber designed for the microscope.

Cells were incubated with 10 $\upmu$M latrunculin-B (Sigma-Aldrich) for 30 min or 16 $\upmu$M nocodazole 45 min to induce actin and microtubule depolymerization, respectively. Microtubule dynamics perturbation was performed incubating the cells with 10 nM vinblastine sulfate (Sigma-Aldrich) for 30 min. Cell spreading was not substantially affected by the drug treatments, as assessed by inspecting the cells’ areas in trasmission images of the samples before and after the incubation time.

Mitochondria were labeled adding 100 nM MitoTracker Deep Red FM (Invitrogen) to the incubation medium.

### Microscopy

Superresolution images were acquired in a Zeiss LSM980 confocal microscope (Carl Zeiss) equipped with the AiryScan 2 detector using a Plan-Apochromat 63x oil immersion objective (NA = 1.4). EGFP-XTP and MitoTracker Deep Red FM were excited with solid diode lasers of 488 nm and 639 nm, respectively. Images were acquired sequentially by frame with pixel size of $\sim$ 40 nm. Raw files were deconvoluted using the standard processing routine from the Zen software.

Confocal images were acquired in FV1000 Olympus confocal microscope (Olympus Inc). EGFP-XTP and MitoTracker Deep Red FM were observed using a multi-line Ar laser tuned at 488 nm and a 635 nm solid diode laser as excitation sources, respectively. The laser’s light was reflected by a dichroic mirror (DM405/488/543/635) and focused through an Olympus UPlanSApo 60× oil immersion objective (NA = 1.35) onto the sample. Fluorescence was collected by the same objective and split into two channels set to collect photons in the range 505–525 nm (EGFP) and 655–755 nm (MitoTracker Deep Red FM). Fluorescence was detected with photomultipliers set in the photon-counting detection mode. Time-lapse images were acquired at a speed of (0.1–1.96 frames/s). Pixel size: 0.055–0.276 $\upmu$m.

### Image analysis and mitochondria tracking

Airyscan-SR images were analyzed using Fiji plugin Mitochondria Analyzer^51^. This software enables quantitative analysis of mitochondrial morphology by means of adaptive thresholding of the image for the correct extraction of mitochondrial objects and a subsequent analysis of the mitochondrial network. Prior to the analysis, the images were preprocessed manually as explained in Supplementary Section 4.

Images of mitochondria were then binarized with the plugin using default settings except from the local-based thresholding method options. These parameters were determined to accomplish an optimal thresholding in a representative set of images of all conditions.

Still, we found that the algorithm resulted in many spurious small mitochondria. For this reason, we filtered objects smaller than 0.3 $\upmu$m$^2$, which corresponded approximately to the maximum size of the spurious dots. After filtering the data, we obtained the number of mitochondria per cell (N), the median area per mitochondrion per cell and the total mitochondrial area per cell. The mitochondrial cell coverage, i.e. fraction of cell area occupied by mitochondria, was obtained by dividing total mitochondrial area by the cell area, which was calculated as explained in Supplementary Section 4. Between 11 and 16 cells were analysed for each condition, a summary of the results can be found in Supplementary Table S2.

Confocal images of individual elongated mitochondria were analyzed using Fiji plugin JFilament^56^. This plugin uses open active contours to segment and track 2D filaments from fluorescence microscopy images. Mitochondria shapes needed to be manually initialized; these shapes were then deformed by the plugin and a set of digitized two-dimensional coordinates representing the organelle curvilinear shape was obtained for each images frame. Between 154–254 mitochondria from 8–40 cells for each condition were segmented and analyzed.

We tracked individual mitochondria throughout the time-lapse movies for temporal analyses. Due to photobleaching, organelle out of focus movement or collisions, not every mitochondria could be tracked throughout the time-lapse movies. However, a total of 49–99 mitochondria were correctly tracked, during 50–150 frames.

### Determination of mitochondria width

Mitochondria width were determined by fitting a gaussian function to the mitochondrial transversal intensity profile for both Airyscan-SR (N = 21) and confocal (N = 17) images of control cells using Plot Profile tool in FIJI. Width was computed as $2\sigma$. Similar values were obtained for both kind of images: $328 \pm 52$ nm and $318\pm 53$ nm for Airyscan-SR or confocal images, respectively. Results are expressed as mean $\pm$ std.

### Mitochondria-microtubules orientation analysis

Mitochondria-microtubule orientation analysis was performed on 10 $\times$ 10 $\upmu$m$^2$ ROIs generated from 2D Airyscan-SR images of EGFP-XTP and MitoTracker Deep Red in *X. laevis* melanophore cells using FIJI. First, mitochondria and microtubules images were synchronized for ROIs selection. Very thin cells protrusions were avoided since they have a predominant orientation that would bias the results. Orientation angles of mitochondria and microtubules in the same ROI were obtained with the Directionality tool in FIJI (https://imagej.net/plugins/directionality). Special care was taken for the analysis of nocodazole treated cells: only cell regions that presented both mitochondria and microtubules were used for the orientation analysis. Between 3–12 ROIs per cell, and 3–9 cells per condition, were analyzed. Microtubules versus mitochondria orientation angles running from − 90$^{\circ }$ to 90$^{\circ }$ were plotted and the data points were compared to a linear regression with slope equal to 1 and 0 intercept (identity). The root mean square error (RMSE) was used to quantify the dispersion of the data from the identity and thus represents an estimate of the differences in the local orientation of mitochondria and microtubules.

### Determination of mitochondrial length and apparent persistence length

We computed the length of an individual mitochondrion integrating the curvilinear coordinate along the recovered shape:

1 $$\begin{aligned} L=\sum _{k=1}^{n_{points}} \Delta s_k, \end{aligned}$$

where $\Delta s_k$ was calculated as:

2 $$\begin{aligned} \Delta s_k = \sqrt{(x_{k+1} - x_k)^2 + (y_{k+1} - y_k)^2} \end{aligned}$$

with ${x_k,y_k}$ the set of cartesian coordinates recovered with the JFilament plugin described above.

Cartesian coordinates were transformed to curvilinear coordinates as follows:

3 $$\begin{aligned} s_k=\sum _{j=1}^{k} \Delta s_j \end{aligned}$$

4 $$\begin{aligned} \theta _k = tan^{-1} \Big (\frac{y_{k+1} - y_k}{x_{k+1} - x_k}\Big ), \end{aligned}$$

where $s_k$ and $\theta _k$ are the arc length and the tangential angle of the recovered curvilinear shape at $s=s_k$, respectively.

The apparent persistence length ($L_p^*$) of mitochondria was computed from the fit of an exponential decay function to the ensemble average of the correlation of the tangent angle $\theta (s)$^58^ using a non-linear least squares fitting routine in Python:

5 $$\begin{aligned} \Big < \cos \big (\theta (s)-\theta (s_0)\big )\Big > = A e^{\frac{-(s-s_0)}{2L_p^*}}. \end{aligned}$$

The parameter $s_0$ is a cutoff distance that takes into account the organelle thickness and *A* represents a correction to the maximum correlation at $s = s_0$. We considered $s_0=0.8 \upmu$m in the ulterior analyses.

In order to have enough data to compute the ensemble average of the correlation of the tangent angle, for each condition we considered mitochondria longer than the corresponding median length and used these lengths as upper cutoff for the fitting of Eq. (5).

The error of the apparent persistence length was computed as the maximum dispersion obtained for the fitted parameter $L_p^*$ for different cutoffs values. Since median lengths of most conditions were in the interval 2–3 $\upmu$m, we computed $L_p^*$ for data up to 2 $\upmu$m, median length and for 3 $\upmu$m. The reported value of the persistence length corresponds to average of these 3 values and the error to interval given by the different values. The values were considered significantly different if the error intervals did not overlap.

### Mitochondrial shape classification

Mitochondria were classified as rod-like, smile-like or snake-like, following the pipeline described in^45^. Briefly, if the mitochondrion shape could be successfully fitted by a linear regression, it was classified as rod-like. Else, $\theta (s)$ was approximated by a Fourier decomposition of 5 modes:

6 $$\begin{aligned} \theta (s) = \sqrt{\frac{2}{L}} \sum _{k=1}^N a_n \cos (\frac{n \pi }{L}s) ), \end{aligned}$$

where the modes amplitudes were calculated as

7 $$\begin{aligned} a_n \sim \sqrt{\frac{2}{L}} \sum _{k=1}^N \theta _k \Delta s_k \cos ( \frac{n \pi }{L} s_k^{mid}) \end{aligned}$$

with $$L=\sum _{k=1}^N \Delta s_k$$ and $$s_k^{mid}=\sum _{j=1}^{k-1} \Delta s_j + 1/2\Delta s_k$$.

A mitochondrion was classified as smile-like if the first mode $a_1>a_i$, with $i=2 \ldots 5$. In any other case, it was classified as snake-like.

### Computation of the shape fluctuations rate

For each mitochondrion in an image sequence, we calculated the curvature $d\theta (s,t)/ds$ using **numpy** function gradient. Then, we averaged the curvature for each frame and computed its autocorrelation function (ACF) using autocorr function from **Pandas** library. The ACF data was then fitted with an exponential decay function and the rate of shape fluctuations was defined as the inverse of the obtained characteristic decaying time.

### Mitochondria mobility analysis

In order to assess the mobility of mitochondria we obtained the movement of their center of mass (CM) throughout the movie and computed D, the net distance explored by the organelle, as:

8 $$\begin{aligned} D = \sqrt{ \Delta X_{CM}^2 + \Delta Y_{CM}^2} \end{aligned}$$

with $\Delta X_{CM}= X_{Max}-X_{Min}$ and $\Delta Y_{CM}= Y_{Max}-Y_{Min}$, as illustrated in Fig. 3c. We quantify the mobility as the quotient between D and the total duration (in minutes) of the tracked CM trajectory:

9 $$\begin{aligned} mobility =\frac{D}{t_{total} }. \end{aligned}$$

### Statistical analysis

The results are expressed as median ± standard error, when not specified otherwise. The standard error of the estimator was calculated through the nonparametric bootstrap procedure in which we obtain new samples of the same size by randomly sampling (with replacement) from the observed data to approximate the population. We computed the median for each bootstrap sample to generate bootstrap sampling distributions and obtained the variance $\sigma ^2$ to assess the standard error^65^. We used 1000 bootstrap repetitions.

We performed a custom-made^50^hypothesis test to test if the medians of different data groups were significantly different. The P-values are obtained as follows:

10 $$\begin{aligned} P \text{-value } =2\left[ 1-F\left( \frac{|{\text {med}}(\textrm{g} 1)-{\text {med}}(\textrm{g} 2)|}{\sqrt{\sigma ^2(\mathrm {~g} 1)+\sigma ^2(\mathrm {~g} 2)}}\right) \right] , \end{aligned}$$

where F is the standard normal distribution and g1 and g2 represent the data groups. Data sets were considered significantly different when $P<0.05$.

In the case of means (junctions data in Supplementary Table S2), we used an ANOVA one-way test. Data sets were considered significantly different when $P<0.05$.

A non-linear least squares method was used in the fittings. The statistical analysis, curve fitting and plots were performed using Python libraries.

## Supplementary Information

Supplementary Information.

Supplementary Video S1.

Supplementary Video S2.

Supplementary Video S3.

Supplementary Video S4.

Supplementary Video S5.

Supplementary Video S6.

Supplementary Video S7.

Supplementary Video S8.

Supplementary Video S9.

Supplementary Video S10.

## Data Availability

The data that support the findings of this study, as well as the custom-written Python routines are available from the corresponding authors upon reasonable request.
