# Author Correction: Mitochondrial cellular organization and shape fluctuations are differentially modulated by cytoskeletal networks

**DOI:** 10.1038/s41598-023-34566-1

**Published:** 2023-05-09

**Authors:** Agustina Belén Fernández Casafuz, María Cecilia De Rossi, Luciana Bruno

**Affiliations:** 1 CONICET-Universidad de Buenos Aires, Facultad de Ciencias Exactas y Naturales, Instituto de Cálculo (IC), 1428 Buenos Aires, Argentina; 2 CONICET-Universidad de Buenos Aires, Facultad de Ciencias Exactas y Naturales, Departamento de Química Biológica, Instituto de Química Biológica (IQUIBICEN), 1428 Buenos Aires, Argentina; 3 Consejo Nacional de Investigaciones Científicas y Técnicas, Buenos Aires, Argentina

Correction to: *Scientific Reports* 10.1038/s41598-023-31121-w, published online 11 March 2023

In the original version of this Article, María Cecilia De Rossi was omitted as a corresponding author. Correspondence and requests for materials should also be addressed to mcecilia.dr@gmail.com.

The original Article has been corrected.

