# Supplementary material for: Mitochondrial cellular organization and shape fluctuations are differentially modulated by cytoskeletal networks
Source: Sci Rep. 2023 Mar 11;13:4065. doi: 10.1038/s41598-023-31121-w (PMC10008531; doi:10.1038/s41598-023-31121-w)
Supplement: Supplementary file 1 — Supplementary Information. [file 41598_2023_31121_MOESM1_ESM.pdf]

Supplementary information

Mitochondrial cellular organization and shape fluctuations are differentially modulated by cytoskeletal networks

Agustina Belén Fernández Casafuz[1], María Cecilia De Rossi [2,3,+] , and Luciana Bruno[1,3,*]

[1]CONICET - Universidad de Buenos Aires, Facultad de Ciencias Exactas y Naturales, Instituto de Cálculo (IC), Buenos Aires, 1428, Argentina
[2]CONICET - Universidad de Buenos Aires, Facultad de Ciencias Exactas y Naturales, Departamento de Química Biológica, Instituto de Química Biológica (IQUIBICEN), Buenos Aires, 1428, Argentina
[3]Consejo Nacional de Investigaciones Científicas y Técnicas, Argentina
+mcecilia.dr@gmail.com
*lucianabrun@gmail.com

The Supplementary information contains the following Sections:

1. Calibration of microtubules depolymerization with nocodazole
2. Microtubule network organization after drugs treatments
3. Mitochondria cellular distribution and morphology in control and cytoskeleton impaired cells
4. Characterization of mitochondrial network using the Fiji plug-in Mitochondria Analyzer
5. Determination and distribution of mitochondria lengths
6. Mitochondria apparent persistence lengths (L_p^*) and shape populations
7. Analysis of mitochondria-microtubules coordinated lateral motion
8. Mitochondria mobility and rate of shape fluctuations
9. Description of Supplementary Videos
10. References

1. Calibration of microtubules depolymerization with nocodazole

Since microtubules have been reported to be essential to mitochondrial distribution and motion (see for instance References [1,2]), we wanted to minimally affect the microtubule network to capture subtle variations in the organization and mobility of mitochondria, which allowed us to compare with the contributions of the other cytoskeleton filaments. Therefore, our aim was not to disrupt the complete microtubule network but to produce small perturbations to this cytoskeletal architecture. To this end, we used the following protocol.

Cells were incubated with 16 uM nocodazole at room temperature for 45 min. Airyscan-SR images were recorded before the addition of the drug and every 15 minutes after its use for 45 min. Supplementary Fig. S1a shows that the microtubules network is partially depolymerized during the indicated time window.

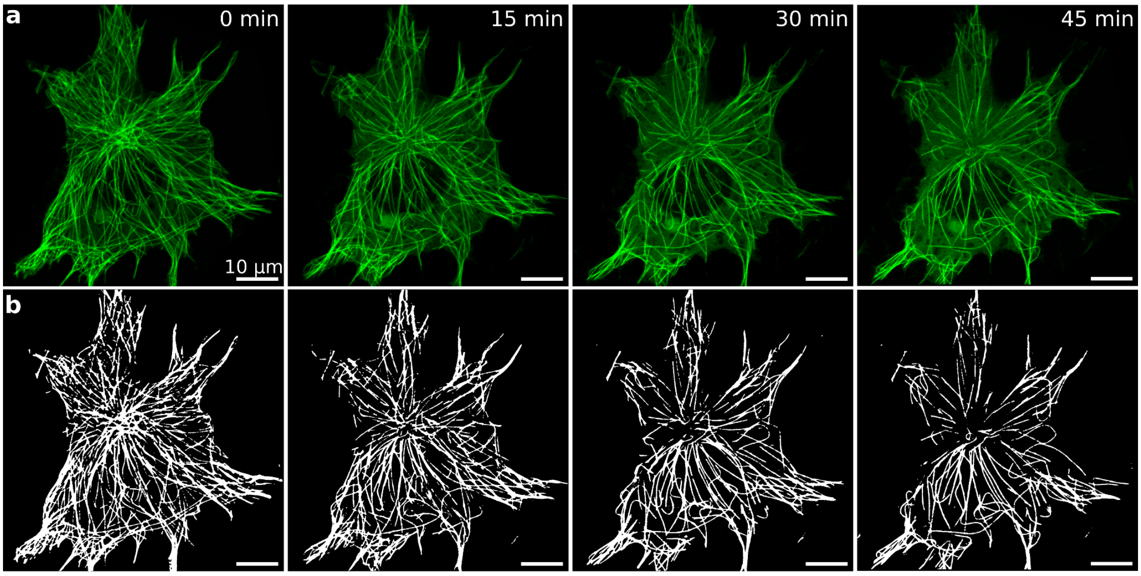

Supplementary Fig. S1. Depolymerization of microtubules during nocodazole treatment. (**a**) Representative Airyscan-SR image of *X. laevis* melanocyte expressing EGFP-XTP (green: microtubules) acquired before and during the incubation with nocodazole 16 µM. (**b**) Binary images of the microtubules network after applying local thresholding with Mitochondria Analyzer plug-in.

To quantify the percentage of depolymerization of these filaments network we used the Fiji plug-in Mitochondria Analyzer[3]. Even though it was conceived as a mitochondrial morphology analyzer, it can also be used to extract the microtubule network successfully and obtain some pertinent parameters to compare the network at different times. We converted the Airyscan-SR images of whole cells to 8-bit, defined a ROI (region of interest) around the selected cell with Fiji's Freehand selection and cleared the outside region, prior to applying the 2D threshold method (Supplementary Fig. S1b). We compared the Total Area occupied by microtubules in the cell at different times during nocodazole incubation and found that, compared with the network before the treatment, the area decreases with time as is illustrated in Supplementary Table S1. To

confirm that the decrease in the area occupied by the microtubules (proportional to the total intensity in the binarized image) was due to a reduction in the number of filaments induced by nocodazole treatment and to rule out that it was caused by photobleaching phenomena, we verified that the intensity values did not vary significantly between the images.

time (min)	total cell area (%)				
	cell 1	cell 2	cell 3	cell 4	cell 5
0	100	100	100	100	100
15	76	-	-	-	-
30	68	-	-	-	-
45	55	79	71	57	88

Supplementary Table S1. Quantification of the total cell area occupied by the microtubules network during nocodazole treatment. The results reported for cell 1 correspond to Supplementary Fig. S1.

2. Microtubule network organization after drugs treatments

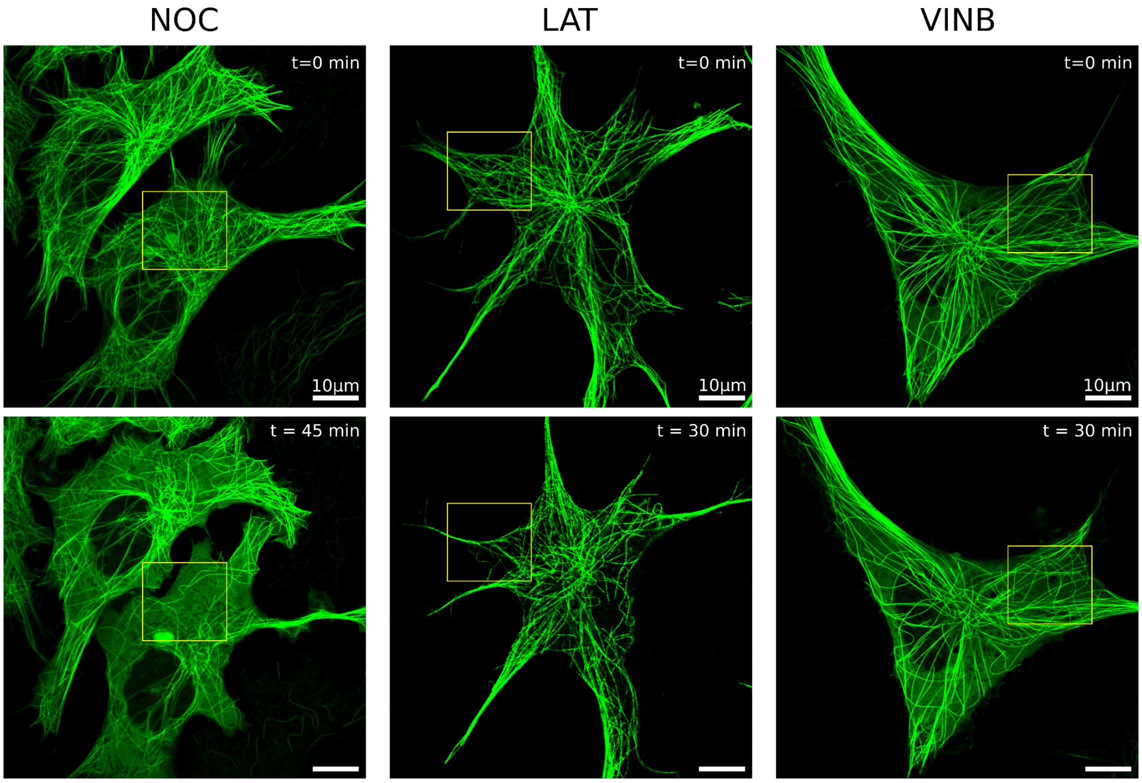

Supplementary Fig. S2. Microtubule network organization before and after different drug treatments that disturb the cytoskeleton. Representative Airyscan-SR image of *X. laevis* melanocyte expressing EGFP-XTP (green: microtubules) acquired before (top panel) and after (bottom panel) the incubation time with nocodazole (NOC), latrunculin-B (LAT) or vinblastine (VINB). Dotted squares indicate representative cellular regions where modifications of the microtubule network are visualized. Note the reduction in the number of microtubules in the nocodazole-treated cell, the condensation of microtubules in thinner cellular projections after latrunculin-B treatment and the increase of microtubules curvatures with vinblastine.

3. Mitochondria cellular distribution and morphology in control and cytoskeleton impaired cells

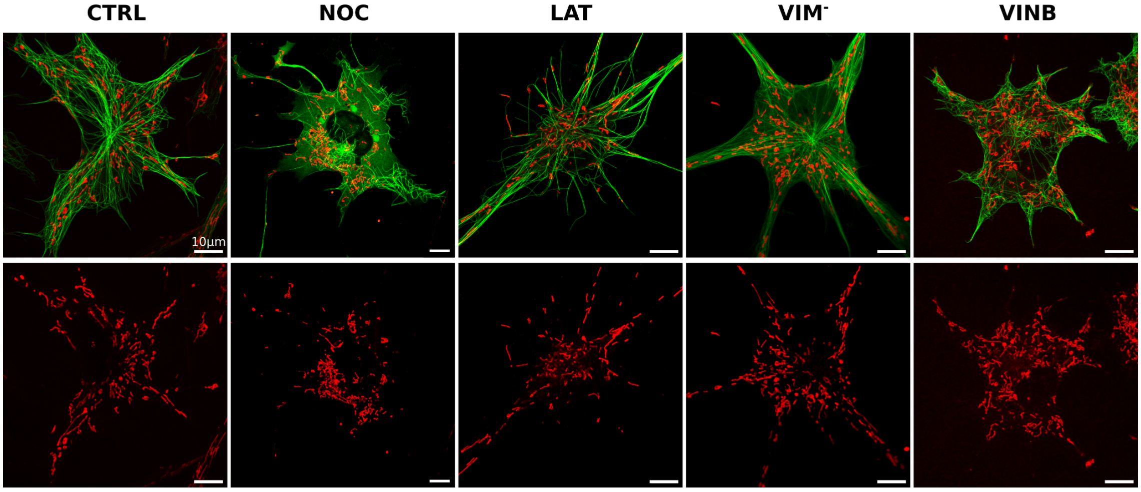

Supplementary Fig. S3. Distribution and morphology of mitochondria after cytoskeleton perturbation. Representative Airyscan-SR images of *X. laevis* melanocyte expressing EGFP-XTP (green: microtubules) and incubated with MitoTracker Deep Red FM (red: mitochondria) (top panel). Cells were registered in control condition (CTRL) and after treatment with nocodazole (NOC), latrunculin-B (LAT) or vinblastine (VINB). Cells expressing mCherry-vim(1–138) (VIM^-) were registered 24 h after transfection. To facilitate visualization of the mitochondria, the red channel is shown independently (bottom panel).

4. Characterization of mitochondrial network using Fiji plug-in Mitochondria Analyzer

Prior to the analysis, the images were preprocessed manually. First, images were converted to 8 bits and a cell mask was created using the microtubule (MT) channel to discard mitochondria from neighbouring cells. This was accomplished by saturating the brightness of the MT channel and making a freehand selection region of interest (ROI) following the edges of the cell, since the MT network proves to be a good template for the cell shape as shown in Supplementary Fig. S4. Then, we cleared the outside of the cell ROI in the mitochondria channel.

We also subtracted an average background intensity value to the whole image, calculated as the average of the median intensity measured in five different background ROIs within the cell. This had little impact on mitochondria intensity and proved useful to reduce the apparition of fictitious mitochondria on the cell edges.

Images of mitochondria were binarized with the plugin using default settings except from the local-based thresholding method options. These settings were determined to accomplish an optimal thresholding in a representative set of images of all conditions. Thresholded images were compared to the original one by one to assure that the chondriome was accurately captured by the binarization. Binarized images that failed this inspection were discarded. For each image, a per-mito basis analysis was performed and morphological and network descriptors were extracted: area of mitochondrion (area mito) and junctions per mitochondrion (points where two or more branches meet).

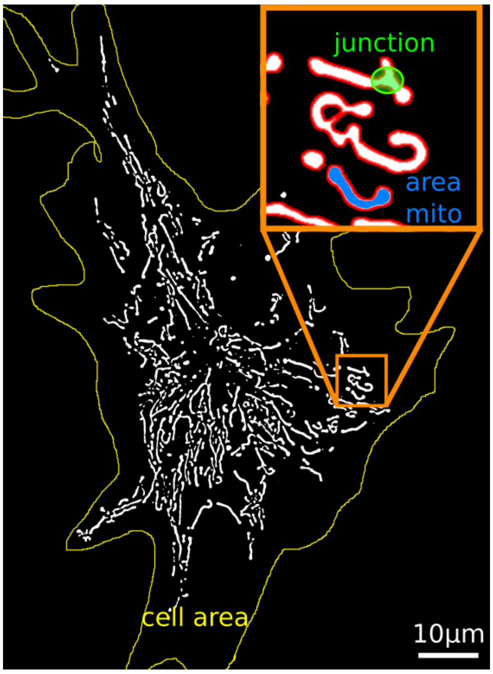

Supplementary Fig. S4. Representative image showing the morphological descriptors used in Supplementary Table S2.

Even though images were preprocessed and parameters were chosen to achieve optimal thresholding, we still found that the algorithm resulted in many spurious small mitochondria. For this reason, we filtered mitochondria smaller than 0.3 μm^2, which corresponded approximately to the maximum size of the spurious organelles. After filtering the data, we obtained the number of mitochondria per cell (N), calculated the median area of mitochondrion per cell and summed over all mitochondrion areas in a cell to obtain the total mitochondrial area.

Mitochondria morphological and networking descriptors obtained for each experimental condition are shown in Supplementary Table S2. To compute the mitochondria cell coverage, we first estimated the explored cell area based on the microtubule network distribution. Supplementary Fig. S5 shows that these filaments network correlates with the morphology and area of the melanocytes. Using the image acquired for microtubules as a reference, a mask was made to delimit the cell area using Fiji software. The cell area was determined from the mask using Area Fraction in the Analyze tool and comparing this magnitude with the image size to obtain the cell area (CA) in micrometres. The mitochondria cell coverage was computed by dividing the total mitochondria area obtained with Mitochondria Analyzer by CA.

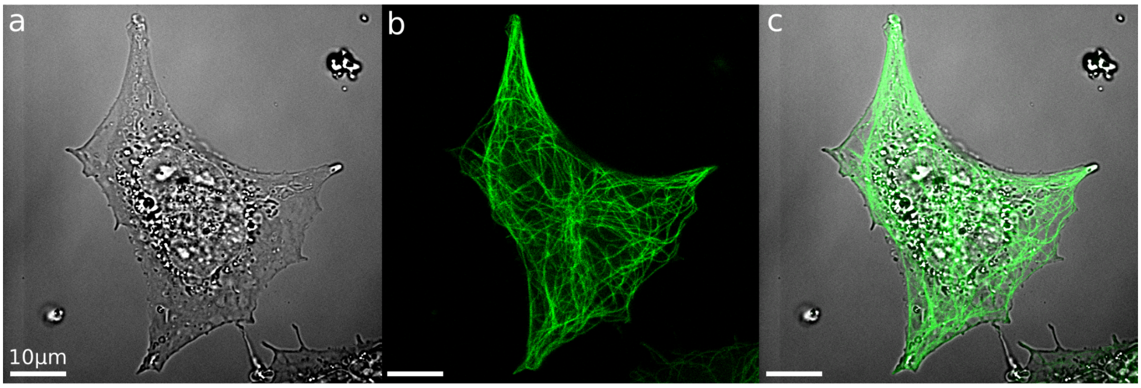

Supplementary Fig. S5. Estimation of melanocyte area. Representative transmission (**a**) and confocal (**b**) images of *X. laevis* melanocyte expressing EGFP-XTP (green: microtubules). (**c**) Merge images demonstrating that the cell area correlates with the distribution of the microtubules network.

Condition	N cells	N mito	mitochondrial cell coverage (%)	area mito (μm^2)	junctions	cell area (μm^2)
CTRL	15	168±40	8.1±0.6	0.72±0.01	0.1±0.4	2190 ±299
NOC	11	116±19	4.5 ±0.1 (*)	0.53±0.01 (*)	0.0±0.2 (*)	2549±854
LAT	11	137±22	6.1±0.7 (*)	0.72±0.02	0.1±0.4	2155±170
VIM$^-$	14	136±12	8.8±0.8	0.74±0.01	0.1±0.5	1697±145
VINB	17	168±15	8.2±0.7	0.69±0.01 (*)	0.2±0.6 (*)	1914±141

Supplementary Table S2. Mitochondria Analyzer results. Morphological and networking descriptors computed were: mitochondria number per cell (N mito), area and junctions per mitochondria (area mito and junctions, respectively) and mitochondria cell coverage described above. The junction data is expressed as mean ± standard deviation. The rest of the data are expressed as median ± standard error. Asterisks denote significant differences (p-value<0.05) between data sets and control condition, as described in Methods.

5. Determination and distribution of mitochondria lengths

The length of the organelles (L) was computed by integrating the curvilinear coordinates along the recovered shape, as explained in Methods. The statistical estimators of the median and mean for control and the different treatments are displayed in Supplementary Table S3, while the distributions are shown in Supplementary Fig. S5. The inspection of the length distributions suggests the presence of different populations. So, we analyzed these data using a Gaussian mixture model[4] that considers the data as a combination of samples obtained from K normal distributions with mean v_k (k = 1, ..., K). The multimodal distribution $f(v)$ is then expressed as:

$$f(v) = \sum_{k=1}^{K} p_k \frac{1}{\sqrt{2\pi\sigma}} e^{-\frac{1}{2}(\frac{v-v_k}{\sigma})^2}$$

where p_k (k = 1, ..., K) represents the relative size of the k-sub-population with

$$\sum_{k=1}^{K} p_k = 1$$

The center v_k of each subpopulation (i.e. mode) is a local maximum of the density distribution. The maximum likelihood estimators of the parameters (σ, v_k, p_k) were obtained using gmdistribution.fit in MATLAB and the model was selected following the Akaike information criterion[5]. The parameters' errors were computed by a bootstrap procedure.

We found that all the conditions but latrunculin-B treatment, could be well fitted by a bimodal distribution (K=2). The length of mitochondria in F-actin depleted cells was better fitted by a model with K=3. The parameters of the model are shown in Supplementary Table S4. Notably, similar results were obtained for control, nocodazole treatment and vimentin IFs disrupted cells: around 90% of the mitochondria belongs to a sub-population with lengths centred in 2.3 μm, while the remaining 10% corresponds to lengths around 6-7 μm. On the other hand, mitochondria in vinblastine-treated cells are shorter: the bimodal distribution is centred in 1.6 and 3.6 μm. Finally, while 80% of the organelles in F-actin depleted cells had lengths similar to the shorter mitochondria in control condition, the rest were significantly longer, with lengths up to 10 times larger.

Condition	N	median (μm)	mean (μm)
CTRL	196	2.60 ± 0.12	2.85 ± 1.77
NOC	154	2.33 ± 0.12	2.67 ± 1.5
LAT	198	2.60 ± 0.19	3.65 ± 3.3
VIM^{-}	252	2.24 ± 0.09	2.77 ± 1.7
VINB	194	1.63 ± 0.07	1.788 ± 0.97

Supplementary Table S3. Quantification of mitochondria length. The data obtained for each experimental condition is expressed as median (or mean) ± standard error (std).

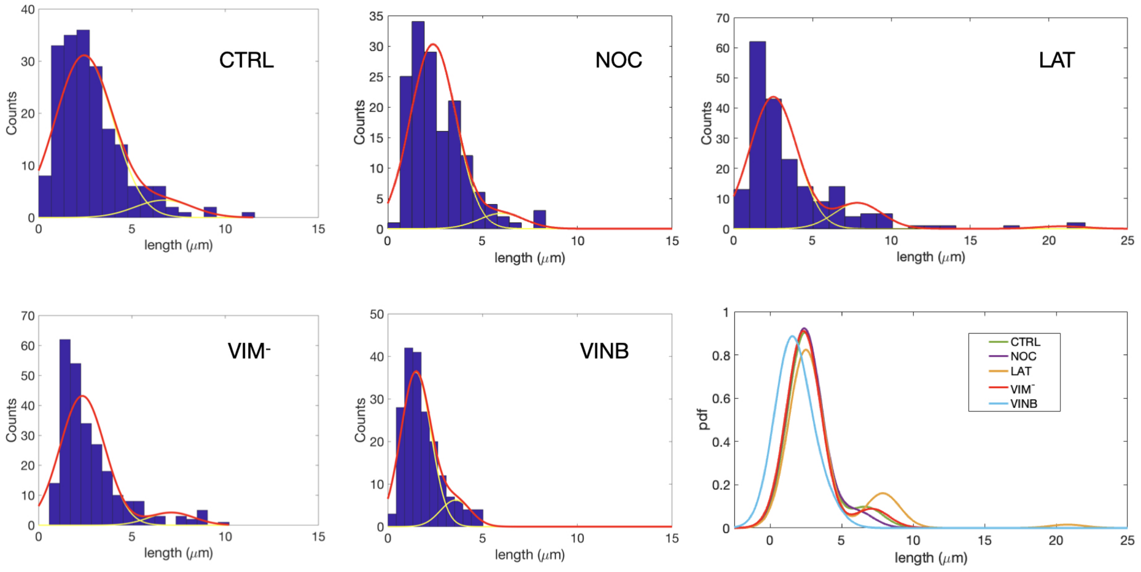

Supplementary Fig. S6. Mitochondria length distributions. The lengths obtained for each experimental condition (blue histograms) were quantitatively analyzed as described above considering a mixture of Gaussian functions (red line) with two or three modes (yellow lines). The gaussian functions for each condition were renormalized considering the corresponding number of data points. The last panel shows the Gaussian mixture probability density functions (pdfs) for all the conditions.

Condition	μ_1 (μm)	μ_2(μm)	μ_3(μm)	f_1	f_2	f_3	σ
CTRL	2.4 ± 0.2	6.7 ± 1.8	0	0.90	0.10	0	1.55
NOC	2.4 ± 0.2	6.0 ± 0.9	0	0.92	0.08	0	1.2
LAT	2.5 ± 0.1	7.9 ± 2.2	21.0± 1.1 (*)	0.82	0.16	0.02	1.5
VIM$^-$	2.3 ± 0.7	7.1 ± 0.1	0	0.90	0.10	0	1.2
VINB	1.55 ± 0.05 (*)	3.6 ± 0.2 (*)	0	0.85	0.15	0	0.8

Supplementary Table S4. Gaussian mixture model parameters. μ_i and σ correspond to the mean position and standard deviation of gaussian i, respectively. Errors were computed following a bootstrap procedure. f_i is the fraction representing the contribution of gaussian i to the overall distribution. Asterisks denote significant differences (p-value<0.05) between data sets and control condition, as described in Methods.

6. Mitochondria apparent persistence lengths (L_p^*) and shape populations.

Related to Fig. 2

Condition	$L_p^* (\mu m)$	rod-like(%)	smile-like(%)	snake-like(%)
CTRL	2.0 ±0.1	44.9	36.7	18.4
NOC	1.7 ± 0.2 (*)	54.5	40.3	5.2
LAT	5.0 ± 0.1 (*)	53.5	34.3	12.1
VIM^-	5.7 ± 0.5 (*)	58.7	34.5	6.7
VINB	2.9 ± 0.4 (*)	72.2	24.7	3.1

Supplementary Table S5. Mitochondria apparent stiffness and shape distribution. L_p^* values obtained for each experimental condition are expressed as median ± standard error (related to Fig. 2c). The values were considered significantly different with respect to the control condition (indicated by asterisks) if the error intervals did not overlap, as described in Methods. The proportion of each mitochondrial population (rod-, smile- and snake-like shapes) corresponding to Fig. 2e are indicated.

7. Analysis of mitochondria-microtubules coordinated lateral motion

To explore whether the motions of mitochondria and their neighbouring microtubules were coordinated, we simultaneously tracked the transversal movement of these structures in confocal images (see Supplementary Video S1) according to the following procedure. First, we merged the images from the red (i.e. mitochondria) and green (i.e. microtubules) channels in Fiji and drew a transversal line in a region that captured both structures (Supplementary Fig. S6a). Using the Fiji plugin KymographBuilder[6], we recovered the time course intensity profiles along the selected line (i.e. kymograph, Supplementary Fig. S6b), which was then splitted in two independent images. Each of these kymographs was then analyzed using a custom made tracking routine[7] that allowed recovering the central coordinates of the transversal position of the mitochondrion and neighbouring microtubules (green dots in Supplementary Fig. S6c). Finally, the recovered trajectories representing their lateral displacement were obtained and the coordinated motion of both structures resulted evident from the observation of this plot (Supplementary Fig. S6d). We randomly sampled similar regions in different movies and in most of the cases we observed a similar behaviour. Other representative examples are shown in Supplementary Fig. S7 and Supplementary Videos S2 and S3.

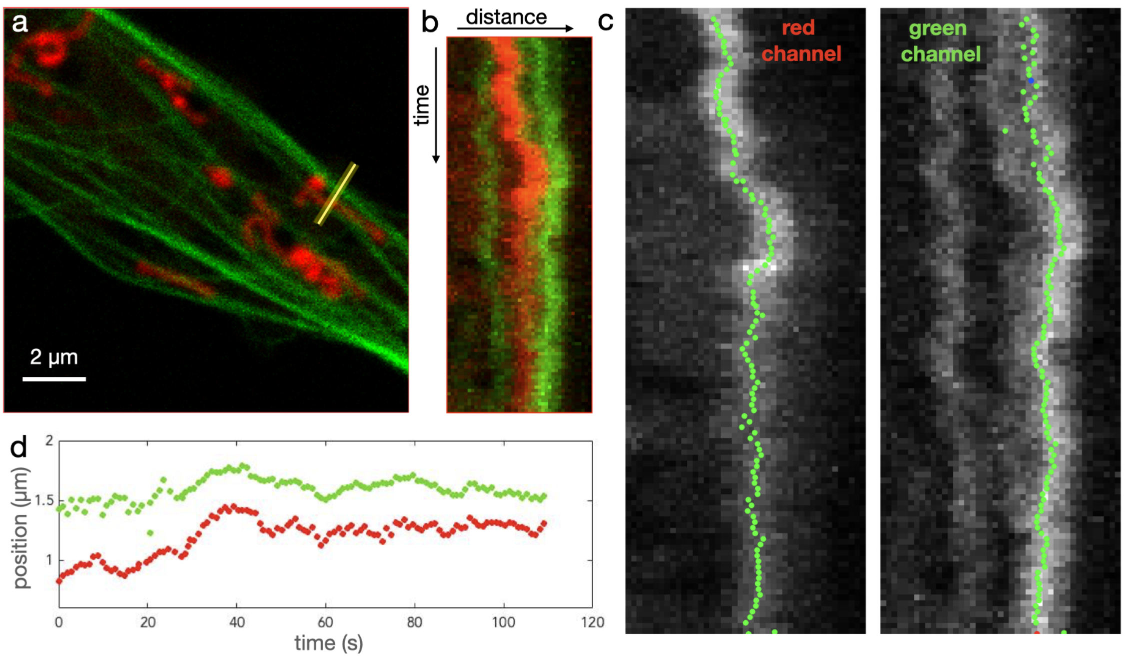

Supplementary Fig. S7. Coordinated lateral motion of a mitochondrion and a neighbouring microtubule. See the text above for further details.

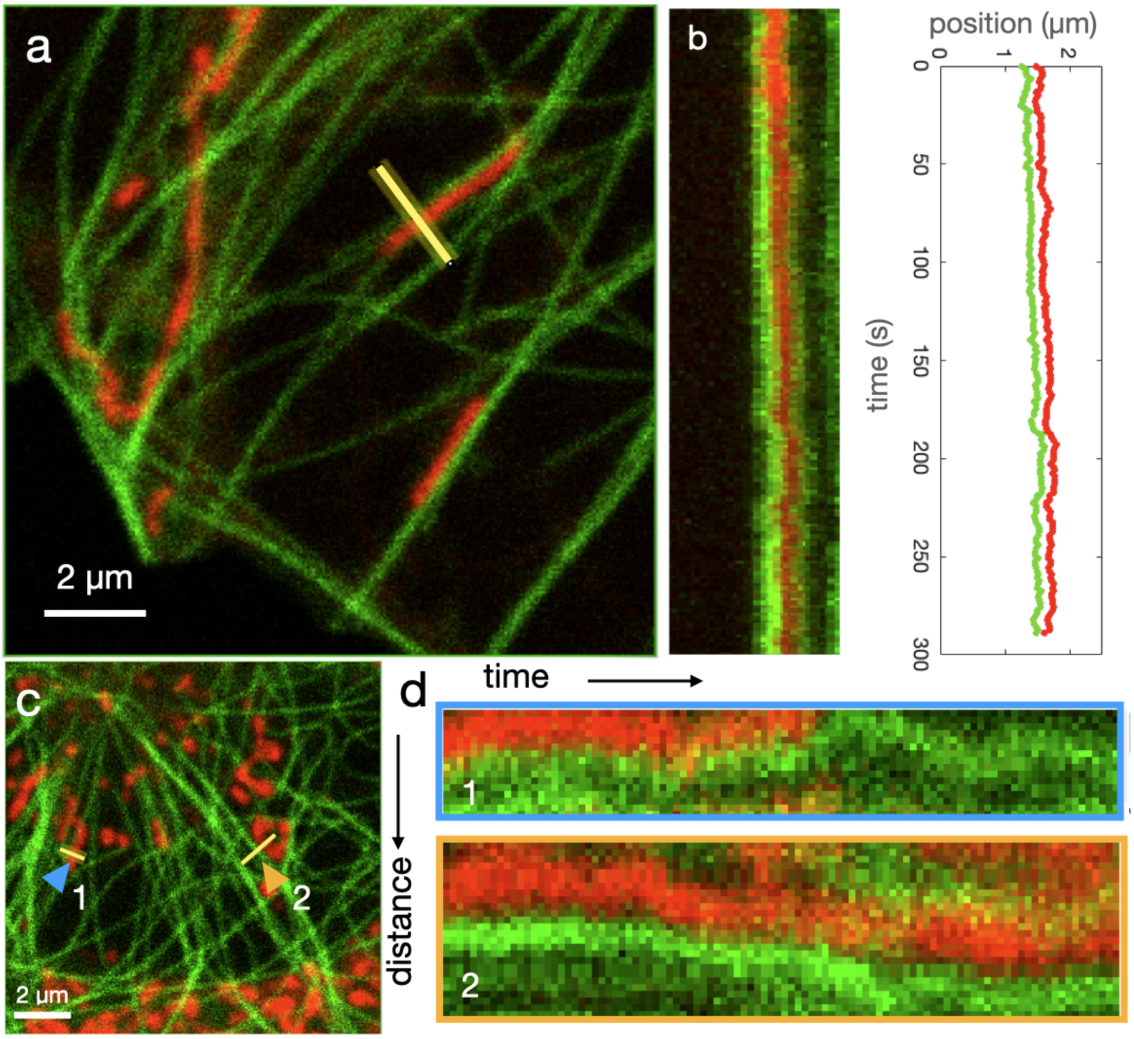

Supplementary Fig. S8. Coordinated lateral motion of a mitochondrion and its neighbouring microtubules: some representative examples. (**a**,**c**) Microtubules (green) and mitochondria (red) merged confocal images. (**b**,**d**) kymographs built from the selected ROIs (yellow lines). Notice the coordinated lateral displacement of both structures in each example.

8. Mitochondria mobility and rate of shape fluctuations

Related to Fig. 3d,f

Condition	shape rate (1/s)	mobility (μm/min)
CTRL	0.08 ± 0.02	0.48 ± 0.04
NOC	0.022 ± 0.007 (*)	0.29 ± 0.04 (*)
LAT	0.10 ± 0.02	1.8 ± 0.2 (*)
VIM$^-$	0.08 ± 0.02	0.50 ± 0.06
VINB	0.08 ± 0.03	0.59 ± 0.07

Supplementary Table S6. Quantification of the mitochondria rate of shape fluctuations and mobility. The data are expressed as median ± standard error. Asterisks denote significant differences (p-value<0.05) between data sets and control condition, as described in Methods.

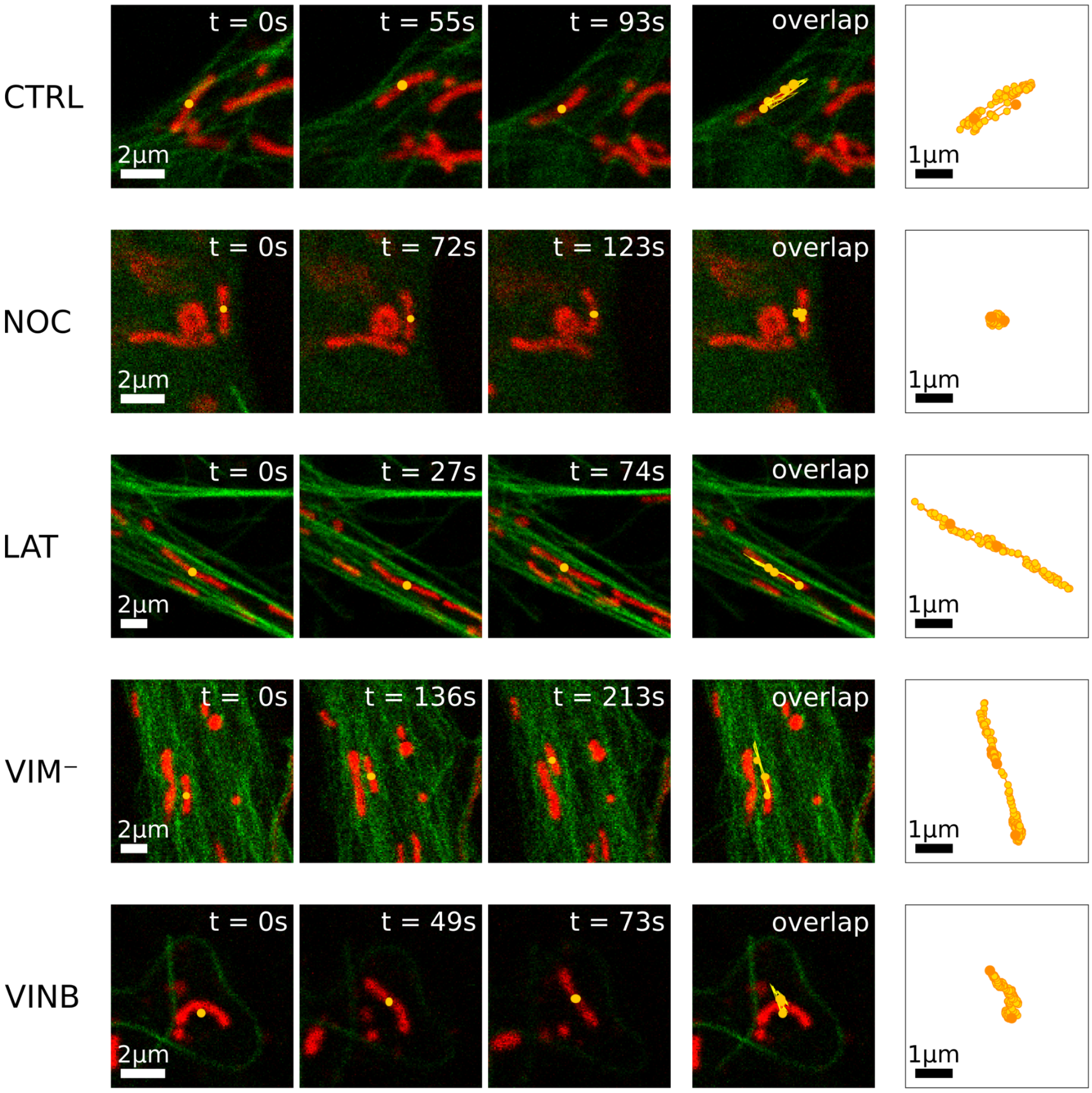

Supplementary Fig. S9. Mitochondria mobility analysis. Representative time-lapse images of mitochondria moving under different cell conditions. The spatial coordinates of the organelles (red) were recovered in each frame of the movies (Supplementary Videos S4-8) to obtain the

trajectory of its center of mass (orange circles). The recovered trajectories are plotted in the right two panels.

9. Supplementary Videos

Supplementary Video S1: *X. laevis* melanocyte expressing EGFP-XTP (green: microtubules) and incubated with MitoTracker Deep Red FM (red: mitochondria) were imaged at 1.02 frames/s (112 frames). Pixel size: 0.055 µm. Related to Supplementary Fig. S7.

Supplementary Video S2: *X. laevis* melanocyte expressing EGFP-XTP (green: microtubules) and incubated with MitoTracker Deep Red FM (red: mitochondria) were imaged at 0.60 frames/s (176 frames). Pixel size: 0.055 µm. Related to Supplementary Fig. S8a.

Supplementary Video S3: *X. laevis* melanocyte expressing EGFP-XTP (green: microtubules) and incubated with MitoTracker Deep Red FM (red: mitochondria) were imaged at 0.60 frames/s (100 frames). Pixel size: 0.055 µm. Related to Supplementary Fig. S8c.

Supplementary Video S4. *X. laevis* melanocyte expressing EGFP-XTP (green: microtubules) and incubated with MitoTracker Deep Red FM (red: mitochondria) were imaged at 0.90 frames/s (200 frames) in CTRL condition. Pixel size: 0.063 µm. Related to Supplementary Fig. S9.

Supplementary Video S5. *X. laevis* melanocyte expressing EGFP-XTP (green: microtubules) and incubated with MitoTracker Deep Red FM (red: mitochondria) were imaged at 0.60 frames/s (150 frames) in NOC condition. Pixel size: 0.063 µm. Related to Supplementary Fig. S9.

Supplementary Video S6. *X. laevis* melanocyte expressing EGFP-XTP (green: microtubules) and incubated with MitoTracker Deep Red FM (red: mitochondria) were imaged at 0.90 frames/s (150 frames) in LAT condition. Pixel size: 0.103 µm. Related to Supplementary Fig. S9.

Supplementary Video S7. *X. laevis* melanocyte expressing EGFP-XTP (green: microtubules) and incubated with MitoTracker Deep Red FM (red: mitochondria) were imaged at 0.82 frames/s (200 frames) in VIM^- condition. Pixel size: 0.101 µm. Related to Supplementary Fig. S9.

Supplementary Video S8. *X. laevis* melanocyte expressing EGFP-XTP (green: microtubules) and incubated with MitoTracker Deep Red FM (red: mitochondria) were imaged at 0.90 frames/s (150 frames) in VINB condition. Pixel size: 0.063 µm. Related to Supplementary Fig. S9.

Supplementary Video S9. *X. laevis* melanocyte expressing EGFP-XTP (green: microtubules) and incubated with MitoTracker Deep Red FM (red: mitochondria) were imaged at 0.60 frames/s (50 frames). Pixel size: 0.055 µm. Mitochondrion showing processive motion is indicated by the yellow arrow.

Supplementary Video S10. *X. laevis* melanocyte expressing EGFP-XTP (green: microtubules) and incubated with MitoTracker Deep Red FM (red: mitochondria) were imaged at 0.90 frames/s (50 frames). Pixel size: 0.063 µm. Mitochondrion showing processive motion is indicated by the yellow arrow.

10. References

[1] Woods, L. C., Berbusse, G. W. & Naylor, K. Microtubules are essential for mitochondrial dynamics–fission, fusion, and motility–in dictyostelium discoideum. Front. cell developmental biology 4, 19 (2016).

[2] Fenton, A. R., Jongens, T. A. & Holzbaur, E. L. Mitochondrial dynamics: Shaping and remodeling an organelle network. Curr. opinion cell biology 68, 28–36 (2021).

[3] Chaudhry, A., Shi, R. & Luciani, D. S. A pipeline for multidimensional confocal analysis of mitochondrial morphology, function, and dynamics in pancreatic β -cells. Am. J. Physiol. Metab. 318, E87–E101 (2020).

[4] Wasserman, L. All of Statistics: A Concise Course in Statistical Inference. Springer-Verlag; New York: 2010.

[5] Schwarz G. Estimating the dimension of a model. Ann Stat. 1978; 62:461–464.

[6] Hadrien Mary, https://imagej.net/plugins/kymograph-builder

[7] https://fernet.exp.dc.uba.ar/
